# Clinical and genetic diagnosis of thirteen Japanese patients with hereditary spherocytosis

**DOI:** 10.1038/s41439-021-00179-1

**Published:** 2022-01-12

**Authors:** Keiko Shimojima Yamamoto, Taiju Utshigisawa, Hiromi Ogura, Takako Aoki, Takahiro Kawakami, Shoichi Ohga, Akira Ohara, Etsuro Ito, Toshiyuki Yamamoto, Hitoshi Kanno

**Affiliations:** 1 Department of Transfusion Medicine and Cell Processing, Tokyo Women’s Medical University, Tokyo, Japan; 2 Institute of Medical Genetics, Tokyo Women’s Medical University, Tokyo, Japan; 3 Tokyo Women’s Medical University Institute for Integrated Medical Sciences, Tokyo, Japan; 4 Department of Pediatrics, Graduate School of Medical Sciences, Kyushu University, Fukuoka, Japan; 5 Department of Pediatrics, Toho University Omori Medical Center, Tokyo, Japan; 6 Department of Pediatrics, Hirosaki University Graduate School of Medicine, Aomori, Japan

**Keywords:** Anaemia, Cytogenetics

## Abstract

Hereditary spherocytosis is the most frequent cause of hereditary hemolytic anemia and is classified into five subtypes (SPH1-5) according to OMIM. Because the clinical and laboratory features of patients with SPH1-5 are variable, it is difficult to classify these patients into the five subtypes based only on these features. We performed target capture sequencing in 51 patients with hemolytic anemia associated with/without morphological abnormalities in red blood cells. Thirteen variants were identified in five hereditary spherocytosis-related genes (six in *ANK1* [SPH1]; four in *SPTB* [SPH2]; and one in each of *SPTA1* [SPH3], *SLC4A1* [SPH4], and *EPB42* [SPH5]). Among these variants, seven were novel. The distribution pattern of the variants was different from that reported previously in Japan but similar to those reported in other Asian countries. Comprehensive genomic analysis would be useful and recommended, especially for patients without a detailed family history and those receiving frequent blood transfusions due to chronic hemolytic anemia.

## Blood disorder: Targeted sequencing helps diagnosis when symptoms vary

Genetic testing can help accurately diagnose people suspected of having a rare blood disorder called hereditary spherocytosis, the clinical symptoms of which often vary. Researchers in Japan led by Keiko Shimojima Yamamoto from Tokyo Women’s Medical University collected blood samples from 51 people with hemolytic anemia associated with/without morphological abnormalities in red blood cells. The researchers sequenced the DNA from five genes known to cause hereditary spherocytosis. They identified genetic defects, some never reported before, in 13 people, providing these individuals with a definitive diagnosis for their blood disorder. The researchers suggest more comprehensive genomic workups could help explain the cause of red blood cell shortages in the remaining study subjects.

## Introduction

Hereditary spherocytosis (HS) is the most frequent cause of hereditary hemolytic anemia^1^. Generally, patients with HS show hemolytic anemia in association with jaundice, reticulocytosis, osmotically fragile spherocytes, gallstones, and splenomegaly^2^. Cholelithiasis and aplastic crises are also common complications^3^. The clinical severity of hemolytic anemia in patients with HS varies widely, ranging from asymptomatic to severe life-threatening hemolytic anemia. Thus, an accurate diagnosis is important to support decision-making pertaining to subsequent treatment strategies, including splenectomy. According to the Online Mendelian Inheritance in Man database (OMIM: https://www.omim.org/), HS is classified into five subtypes associated with five different genes responsible for the deficiency or dysfunction of red blood cell membrane proteins, including ankyrin 1 (*ANK1*; MIM #18200 [SPH1]), β-spectrin (*SPTB*; MIM #616649 [SPH2]), α-spectrin (*SPTA1*; MIM #270970 [SPH3]), band 3 protein (*SLC4A1*; MIM #612653 [SPH4]), and protein 4.2 (*EPB42*; MIM #612690 [SPH5]) (Supplemental Table S1). SPH1, SPH2, and SPH4 are associated with the autosomal dominant (AD) trait, whereas SPH3 and SPH5 are associated with autosomal recessive (AR) traits. Therefore, it is important to obtain an accurate diagnosis for proper genetic counseling. For this purpose, it is important to not only evaluate family history, the clinical course, and physical findings but to also perform laboratory examinations^3^. With the recent development of molecular analysis methods, it has become necessary to detect causative gene variants to obtain a final diagnosis for HS patients.

Recently, we developed an originally designed target capture sequencing (TCS) panel for the precise diagnosis of hemolytic anemia. Here, some of the results obtained with this panel are summarized to clarify the clinical and genetic features of patients with HS in association with the five well-established subtypes, SPH1-5.

## Materials and methods

This study aimed to elucidate the molecular basis of HS in Japanese patients. For this purpose, we enrolled patients with hemolytic anemia, including HS, in accordance with the Declaration of Helsinki, followed by the approval of the Ethics Committee of our institution. After obtaining written informed consent, blood samples were collected from patients. From the attending doctors, we also obtained detailed clinical information, including family histories, clinical courses, and physical findings. Between 2016 and 2018, 51 patients showed clinical histories of hemolytic anemia associated with/without morphological abnormalities in red blood cells according to routine laboratory examinations and were enrolled as the subjects of this study.

In most of the patients, when possible, we first performed additional chemical tests, including the acidified glycerol hemolysis time test, the flow-cytometric osmotic fragility (FCM-OF) test, and the eosin-5′-maleimide (EMA) binding test with a negative direct antiglobulin test as per previously reported methods^4–8^.

Genomic DNA was extracted from the patients’ peripheral blood using a QIAamp DNA extraction kit according to the manufacturer’s instructions (QIAGEN, Hilden, Germany). The Haloplex HS target enrichment system (Agilent Technologies, Santa Clara, CA, USA) was used as the target panel. The target panel was designed using SureDesign (https://earray.chem.agilent.com/suredesign/home.htm) to include all coding exons and intron-exon boundaries of 74 possible candidate genes (Supplementary Table S2). Massive parallel sequencing was performed using the Illumina MiSeq platform (Illumina Inc., San Diego, CA, USA). Raw data were aligned to the GRCh37/hg19 human genome. The generated FASTQ files were imported into SureCall ver3.5 (Agilent Technologies) for variant calling.

The obtained variants were filtered according to the following strategy: (1) variant frequencies were below 1% in 1000G_EAS and ALL (1000 Genomes), HGDV, and dpSNP; (2) synonymous variants were excluded (nonsynonymous variants, variants associated with a frameshift, insertion/deletion variants, and variants in splicing donor/acceptor sites were included); (3) variants with allele frequencies of less than 30% within the total read depth were excluded; and (4) the CADD_phred value was higher than 20 if obtained. Variant information obtained through wANNOVAR (http://wannovar.wglab.org/) was used for curation. The Integrative Genomics Viewer (IGV; https://software.broadinstitute.org/software/igv/) was used for visual evaluation. The final conclusion was reached following the American College of Medical Genetics and Genomics (ACMG) guidelines^9^.

The effect of the splicing site variants was evaluated with in silico software using the Berkeley Drosophila Genome Project (https://www.fruitfly.org/seq_tools/splice.html) and DTU Bioinformatics (http://www.cbs.dtu.dk/services/NetGene2/) databases.

## Results

The 13 variants identified in the genes related to SPH1-5 (*ANK1*, *SPTB*, *SPTA1*, *SLC4A1*, and *EPB42*), together with the clinical data and laboratory test results, are summarized in Table 1. Among the 51 subjects, eight patients (patients 1, 3, 4, 7, 8, 9, 10, and 12) were primarily and clinically suspected of having HS and showed HS-related variants, indicating a 100% detection ratio. On the other hand, HS was primarily not suspected in five other patients who showed HS-related variants.

**Table 1:** Results of this study.

	Patient 1	Patient 2	Patient 3	Patient 4	Patient 5	Patient 6	Patient 7	Patient 8	Patient 9	Patient 10	Patient 11	Patient 12	Patient 13
Subtype	SPH1	SPH1	SPH1	SPH1	SPH1	SPH1	SPH2	SPH2	SPH2	SPH2	SPH3	SPH4	SPH5
Gender	Female	Female	Male	Female	Male	Male	Male	Male	Female	Male	Female	Male	Male
Age	37 y	71 y	6 y	1 y 6 m	2 m	15 y	14 y	16 y	13 y	27 y	62 y	45 y	55 y
Family history	–	–	+	–	+	+	+	–	+	–	–	+	–
Clinical histories													
Splenomegaly	+	+	+	+	–	+	+	+	–	+	–	+	+
Splenectomy	–	–	+	–	–	–	+	–	–	+	–	+	–
Gallstone	+	+	–	–	–	–	+	–	–	–	+	–	–
Blood transfusion	NA	+	+	+	+	–	+	–	–	–	+	–	+
Laboratory testing													
Hb (g/dL) [13 < : male, 12 < : female]	11.7	7.1	13.5	7.9	8.2	12.6	15.6	11.2	11	12.9	5.9	15.6	5.1
MCV (fL) [86~98 fL]	86.8	106.1	80.2	85.2	87.6	95	86.5	84.9	82	88.9	106.9	96	113.2
MCHC (%) [31–35%]	34.6	36.2	37	31.1	34	34.8	35.3	33	35.5	33.5	32.1	35.5	31.6
Reticurocytes (‰) [0.2–2.7‰]	92	55	18.6	248	26	44	16.5	69	17.9	30	34	NA	19.5
LDH (U/L) [240~490 U/L]	162	187	200	535	281	272	246	222	246	240	156	157	286
Haptoglobin (mg/dL) [19–170 mg/dL]	<10	<2	NA	<10	NA	2	43	1	<10	20	37	NA	≦10
Total Bilirubin (mg/dL) [0.2–1.2 mg/dL]	3.7	9.9	0.4	1.8	2.2	3.2	1	6.5	3.8	1.5	3.4	36	1.8
Indirect Bilirubin (mg/dL) [0.2–1.0 mg/dL]	0.6	5.1	0.1	0.8	2.1	2.7	0.6	6	2.6	0.8	3.1	NA	1
RBC morphology	Anysocyte,spherocyte	Elliputocyte, spherocyte, stomatocyte	Codocyte, spherocyte	Anisocyte, spherocyte	Anisocyte, codocyte, stomatocyte	Anisocyte, codocyte	Spherocyte	Spherocyte	Spherocyte	Spherocyte	Anisocyte, codocyte, stomatocyte	Spherocyte	Anisocyte, erythrocyte, polychromatophilic, stomatocyte
Specific examination													
AGLT	150 s	30 min<	NA	NA	30 min<	30 min<	NA	NA	NA	NA	30 min<	NA	45sec
EMA (% of control)	72.7	93	35.8	70.1	67.7	97.3	34.8	32	15.3	NA	106.3	15.8	90.2
FCM-OF(46.0–68.8%)	23.8	NA	10.2	7.2	NA	NA	14.9	17.2	4.2	13.6	NA	1.8	NA
Other findiings	–	–	–	–	–	–	–	–	–	–	–	Spina bifida	Epilepsy, developmental delay, hearing difficulty
Variant information													
Chromosome	Chr8	Chr8	Chr8	Chr8	Chr8	Chr8	Chr14	Chr14	Chr14	Chr14	Chr1	Chr17	Chr15
Gene	ANK1	ANK1	ANK1	ANK1	ANK1	ANK1	SPTB	SPTB	SPTB	SPTB	SPTA1	SLC4A1	EPB42
Genomic cordinate (GRCh37/hg19)													
Start	41655025	41584867	41584808	41584761	41580711	41575626	65259974	65249125	65246577	65239394	158632491	42334875	43507389
End	41655025	41584867	41584812	41584764	41580711	41575626	65259974	65249125	65246577	65239395	158632491	42334875	43507389
HGVS (Coding)	NM_000037.4	NM_001142446.2	NM_000037.4	NM_000037.4	NM_000037.4	NM_000037.4	NM_001024858.4	NM_001024858.4	NM_001024858.4	NM_001024858.4	NM_003126.4	NM_000342.4	NM_000119
Location	Intron 1	Intron 4	Exon 5	Intron 5	Exon 9	exon11	Exon 13	Exon 19	Exon 20	Exon 25	Intron 17	Exon 13	exon3
cDNA change	c.27 + 5G>C	c.427-1G>T	c.382_386del	c.426 + 4_426 + 7del	c.841C>T	c.1204G>A	c.2407del	c.4149dup	c.4339G>T	c.5456_5457delinsTT	c.2464 + 1 G>A	c.1469G>A	c.424G>A
Protein change			p.Lys128Phefs*7		p.Arg281*	p.Glu402Lys	p.Glu803Serfs*17	p.Arg1384Alafs*7	p.Glu1447*	p.Glu1819Val		p.Arg490His	p.Ala142Thr
Type	Splicing	Splicing	Frameshift	Splicing	Nonsense	Missense	Frameshift	Frameshift	Nonsense	Missense	Splicing	Missense	Missense
Status	Hetero	Hetero	Hetero	Hetero	Hetero	Hetero	Hetero	Hetero	Hetero	Hetero	Homo	Hetero	Homo
dbSNP	–	–	–	–	–	rs1350849760	–	rs778901641	–	–	rs774632615	–	rs104894487
Clinvar	–	–	–	–	–	Not reported	–	Not reported	–	–	Likely pathogenic	–	Uncertain significance
SIFT (score)	NA	NA	NA	NA	NA	0.028	NA	NA	NA	NA	NA	0	0
Polyphen2 (score)	NA	NA	NA	NA	NA	1	NA	NA	NA	NA	NA	1	1
CADD_phred	NA	27.5	NA	NA	37	34	NA	NA	38	NA	25	29.6	29.5
ACMG criteria	PS1,PM2,PM4, PM6,PP3,PP4	PM2,PM4, PM6,PP4	PVS1, PM2,PM4	PM2,PM4, PM6,PP4	PVS1, PM2,PM4	PM2, PP3, PP4	PVS1, PM2, PM4	PVS1, PM2, PM6, PP4	PVS1, PM2, PM4	PM2, PM6, PP4	PS1, PM2, PM3, PP4	PM2, PM5, PP3, PP4	PS1, PM2, PM3, PP3, PP4
Interpretation	Pathogenic	Likely pathogenic	Pathogenic	Likely pathogenic	Likely pathogenic	VUS	Pathogenic	Pathogenic	Pathogenic	VUS	Likely pathogenic	Likely pathogenic	Pathogenic
Previous report	Nakanishi et al.^12^ (Ankyrin Nara II)	Novel	Novel	Novel	van Vuren et al.^13^	–	Novel	–	Novel	Novel	–	Novel$	Bouhassira et al.^11(Protein 4.2 NIPPON)^

*y* years; *m* months; *NA* not available; *MCV* mean corpuscular volume; *MCHC* mean corpuscular hemoglobin concentration; *LDH* lactate dehydrogenase; *RBC* red blood cell; [], standard value; _, underbar indicates abnormal finding; *chr* chromosome; *VUS* variant of uncertain significance; *$* a similar variant “R490C” was reported by Dhermy et al.^10^

Some damaging scores could not be calculated because they were splicing or loss-of-function.

In cases of splenectomy, only the data of the patients in the period of post-splenectomy is available.

Although two variants identified in patients 6 and 10 were evaluated as “variants of uncertain significance (VUS)” in accordance with ACMG guidelines, the prediction scores for the variant in patient 6 suggested “damage”, and the variant in patient 10 was quite unique. Thus, we considered these variants to likely to be related to disease occurrence. Seven variants were novel and were not included in any database. Among these variants, the variant in patient 12 was similar to that reported by Dhermy et al.^10^. Three of the variants have been previously reported^11–13^. Four variants were observed in the dbSNP database (https://www.ncbi.nlm.nih.gov/snp/), and one of them was evaluated as likely pathogenic in the ClinVar database (https://www.ncbi.nlm.nih.gov/clinvar/).

The variant identified in patient 4 (c.426 + 4_426 + 7del) has not been reported previously but was found to be similar to the Ankyrin Shiga variant (c.426 + 3_426 + 4insA). Through in silico analyses using two different websites, this insertion was predicted to cause the loss of the donor site (Supplementary Figs. S1, S2). Thus, we concluded that this variant was the likely cause underlying abnormal splicing.

Patient 13 harbored a homozygous *EPB42* variant. Since the parents of patient 13 are first cousins, it is suspected that both parents are heterozygous carriers. Patient 11 also showed a homozygous splicing variant in *SPTA1*; however, consanguinity was not found in this patient’s family history.

Among the 38 patients who showed no pathogenic variants in the five genes, 20 patients were analyzed with the FCM-OF test, and only 5 patients showed low values (data not shown).

## Discussion

Inoue et al. analyzed the genetic backgrounds of Japanese HS patients using sodium dodecyl sulfate–polyacrylamide gel electrophoresis and reported band 3 deficiency (SPH4) in 32% of the patients, spectrin deficiency (corresponding to SPH2 and SPH3) in 15%, protein 4.2 deficiency (SPH5) in 6%, ankyrin deficiency (SPH1) in 2%, combined deficiency in 36%, and no abnormality in 9%^14^. In contrast, Yawata et al. analyzed 60 Japanese patients with HS using a similar method and detected protein 4.2 deficiency (SPH5) in 45% of the patients, band 3 deficiency (SPH4) in 20%, and ankyrin deficiency (SPH1) in 7%, while 28% of the patients had an unknown etiology^15,16^. Genetic variants in *ANK1* were analyzed by Nakanishi et al.^12^, who identified 16 variants in 49 patients with HS, suggesting that *ANK1* variants (SPH1) are not rare in the Japanese population.

Remarkable progress has been made in genomic analyses in the last decade. Next-generation sequencing (NGS) is being extensively used in this field. This has helped to expand our understanding of the genetic heterogeneity associated with HS^17^. Some studies have demonstrated the usefulness of the targeted NGS approach for the investigation of specific subtypes of patients with hemolytic anemia^18–20^. Exome sequencing has also been applied to hereditary hemolytic anemia^21,22^.

To confirm ethnic differences in HS-related variants, Choi et al. reviewed the available reports regarding HS-related mutations in comparison with the results of the present study^20^. In reports from the United States, SPTA1 mutation (SPH3) was found to be the most common^23^. A study in the Netherlands revealed that *SPTA1*, *ANK1*, and *SPTB* (SPH3, SPH1, and SPH2) were ranked as the top three genes with identified variants^13^. In Korea, *SPTB* mutation (SPH2) was found to be the most common, followed by *ANK1* mutation (SPH1)^20^. Another study in Korea reported that 25 patients with HS carried mutations in *ANK1* (SPH1; *n* = 13) or *SPTB* (SPH2; *n* = 12)^24^. A study from China reported that among 23 patients, 13 mutations were observed in *ANK1* (SPH1), while 10 mutations were observed in *SPTB* (SPH2)^25^. Other studies have reported similar observations^26–28^. The distribution of the variants is summarized in Supplementary Table S3.

In this study, *ANK1* variants (SPH1) were found to be the most common, being found in 46% (6/13) of the patients. *SPTB* variants (SPH2) were identified in 31% (4/13) of the patients. Thus, the distribution of the variants was similar to those observed in other Asian countries but was different from those observed in non-Asian countries. Previously, *EPB42* (SPH5) was considered the most common subtype. However, the distribution of HS-related variants observed in this study was different from that identified in a previous study on Japanese patients with HS^15,16^. The reason for this difference is unknown; however, the total number of samples examined in the present study is too small to be compared with this previous study. Thus, the analysis of more samples is necessary to better understand the genetic basis of HS in Japanese patients.

As mentioned above, *ANK1* (SPH1), *SPTB* (SPH2), and *SLC4A1* (SPH4) are related to AD traits, whereas *SPTA1* (SPH3) and *EPB42* (SPH5) are related to AR traits. In this study, variants in AD-related genes were found in 11 patients (85%). As six patients (46%) showed a positive family history, the identified variants were considered to be inherited from the affected ancestors. In comparison, five patients with variants in AD-related genes (45%) had no family history, and it remains unknown whether the identified variants occurred de novo or if they were inherited from nonsymptomatic parents. This is a limitation of this study, as parental analysis was not conducted.

We did not find any genotype-phenotype correlation. The observed severities of the clinical and laboratory findings were variable, even within the subgroups classified in accordance with the gene variants. Similar findings have been reported previously^23,29^. Phenotypic variabilities have been reported in a pair of twins with de novo ANK1 missense variants^30^. Thus, even though we found no clear genotype-phenotype correlation, our results are not contradictory to those reported previously.

Regarding laboratory testing, it is difficult to detect HS using only one method because its clinical phenotypes are widely variable. Therefore, more than one test is generally recommended^31^. Previously, the osmotic fragility test was considered to be the gold standard for HS diagnosis^32^. In this study, we found that only the results obtained from FCM-OF matched the results of TCS in this study; all patients with pathogenic HS variants who underwent the FCM-OF test showed low FCM-OF values, but only 5 of 20 patients without HS variants showed low values in the FCM-OF test. This indicates that the FCM-OF test presents high sensitivity but low specificity. Because the combination of the FCM-OF and EMA tests can correctly diagnose 100% of patients^33^, FCM-OF may be the best possible single test for the diagnosis of HS, followed by EMA.

In this study, 38 patients showed no pathogenic variants in the five analyzed genes related to HS. However, we cannot completely deny the possibility of those variants in the analyzed five genes may have been overlooked due to analytical limitations, even though we conducted a comprehensive genomic analysis using next-generation sequencing. Because hemolytic anemia is a heterogeneous entity and the clinical diagnosis of hereditary hemolytic anemia is often inaccurate due to overlapping phenotypes^18^, variable genomic backgrounds are suspected to exist in patients without pathogenic variants in known HS genes. Therefore, molecular diagnosis may help to predict the future prognosis of young patients with HS, along with genetic counseling^29^. Comprehensive genomic analysis would be useful and recommended, especially for patients without a detailed family history and patients with chronic hemolytic anemia receiving frequent blood transfusions.

## Supplementary information

Supplemental Figure S1, 2

Supplemental Table S1

Supplemental Table S2

Supplemental Table S3
