# Supplementary material for: Clinical and genetic diagnosis of thirteen Japanese patients with hereditary spherocytosis
Source: Hum Genome Var. 2022 Jan 12;9:1. doi: 10.1038/s41439-021-00179-1 (PMC8755803; doi:10.1038/s41439-021-00179-1)
Supplement: Supplementary file 1 — Supplemental Figure S1, 2 [file 41439_2021_179_MOESM1_ESM.pdf]

Supplemental Figure S1. In-silico analysis for predicted splice site

http://www.fruitfly.org/seq_tools/splice.html

Wild type

Splice site predictions for 1 sequence with donor score cutoff 0.40, acceptor score cutoff 0.40 (exon/intron boundary shown in larger font):

Donor site predictions for 10.42.3.122.160411.0 :

Start	End	Score	Exon Intron
7	21	0.77	ccaaataggtatgtcc
61	75	0.69	gaatgagggtgtgcag
251	265	0.98	cctccagggtgagagg
427	441	0.99	cacagaaggtaagtac
490	504	0.66	gcttcagggtgacacc

Variant

Splice site predictions for 1 sequence with donor score cutoff 0.40, acceptor score cutoff 0.40 (exon/intron boundary shown in larger font):

Donor site predictions for 10.42.0.144.160423.0 :

Start	End	Score	Exon Intron
7	21	0.77	ccaaataggtatgtcc
61	75	0.69	gaatgagggtgtgcag
251	265	0.98	cctccagggtgagagg
486	500	0.66	gcttcagggtgacacc

In comparison with the wild type sequence, the normal splicing donor site (red rectangle) is disappeared in the variant sequence.

Supplemental Figure S2. In-silico analysis for predicted splice site

http://www.cbs.dtu.dk/services/NetGene2/

Wild type

```
********************** NetGene2 v. 2.4 ************************


The sequence: sequence1 has the following composition:

Length: 780 nucleotides.
26.5% A, 20.1% C, 29.9% G, 23.5% T, 0.0% X, 50.0% G+C

Donor splice sites, direct strand
---------------------------------
               pos 5'->3'  phase strand  confidence  5'      exon intron    3'
                  258         0      +         0.49      TGTCCTCCAG^GTGAGAGGAC
                  434         0      +         0.93      AGCCACAGAA^GTAAGTACCT H
```

Variant

```
********************** NetGene2 v. 2.4 ************************


The sequence: sequence1 has the following composition:

Length: 776 nucleotides.
26.4% A, 20.2% C, 29.9% G, 23.5% T, 0.0% X, 50.1% G+C

Donor splice sites, direct strand
---------------------------------
               pos 5'->3'  phase strand  confidence  5'      exon intron    3'
                  258         0      +         0.49      TGTCCTCCAG^GTGAGAGGAC
```

In comparison with the wild type sequence, the normal splicing donor site (red rectangle) is disappeared in the variant sequence.
