# Supplementary material for: Clinical and genetic diagnosis of thirteen Japanese patients with hereditary spherocytosis
Source: Hum Genome Var. 2022 Jan 12;9:1. doi: 10.1038/s41439-021-00179-1 (PMC8755803; doi:10.1038/s41439-021-00179-1)
Supplement: Supplementary file 2 — Supplemental Table S1 [file 41439_2021_179_MOESM2_ESM.pdf]

Supplemental Table S1. Classification of hereditary spherocytosis in OMIM

MIM No.	Phenotype	Symbol	Location	Gene	Inheritance	Protein
#18200	Spherocytosis type 1	SPH1	8p11.21	*ANK1*	AD, AR	ankyrin 1
#616649	Spherocytosis type 2	SPH2	14q23.3	*SPTB*	AD	β-spectrin
#270970	Spherocytosis type 3	SPH3	1q23.1	*SPTA1*	AR	α-spectrin
#612653	Spherocytosis type 4	SPH4	17q21.31	*SLC4A1*	AD	band 3
#612690	Spherocytosis type 5	SPH5	15q15.2	*EPB42*		protein 4.2

AD, autosomal dominant; AR, autosomal recessive
