# Supplementary material for: Clinical and genetic diagnosis of thirteen Japanese patients with hereditary spherocytosis
Source: Hum Genome Var. 2022 Jan 12;9:1. doi: 10.1038/s41439-021-00179-1 (PMC8755803; doi:10.1038/s41439-021-00179-1)
Supplement: Supplementary file 3 — Supplemental Table S2 [file 41439_2021_179_MOESM3_ESM.pdf]

Supplemental Table S2. The list of the genes included in the target capture sequencing panel

Genes related to enzyme	Genes related to membrane	Gene related to congenital dys-erythropoietic anemia	Genes related to hemo-chromatosis	Gene related to TTP	Genes related to atypical HUS	Others	Genes related to other phenotypes
ADA	*EPB42*	*SEC23B*	*HFE*	*ADAMTS13*	*C3*	*ATP11C*	*ADORA2B*
ALDOA	*SLC4A1*	*CDAN1*	*HFE2*		*CD46*	*COL4A1*	*ULK1*
AK1	*SPTA1*	*C15orf41*	*HAMP*		*CFH*	*PIGA*	*BECN1*
ENO1	*SPTB*	*KLF1*	*TFR2*		*CFI*	*UGT1A1*	*PIK3C3*
G6PD	*ANK1*	*KIF23*	*SLC40A1*		*CFHR1*	*ABCG8*	*AMBRA1*
GPI	*GYPC*		*FTH1*		*CFHR3*	*ATP7A*	*PRKAA1*
GPX1	*EPB41*				*CFB*	*ATP7B*	*ATG9A*
GSR	*STOM*				*THBD*	*GATA1*	*TBC1D5*
GSS	*RHAG*				*CFHR2*		*BNIP3L*
GCLC	*PIEZO1*				*CFHR4*		*BNIP3*
HK1	*SLC2A1*				*CFHR5*		*FUNDC1*
NT5C3A	*KCNN4*				*DGKE*		
PKFM	*ABCG5*				*PLG*		
PGD							
PGK1							
PKLR							
TPI1							

TTP, thrombotic thrombocytopenic purpura; HUS, hemolytic-uremic syndrome
