# Supplementary material for: Clinical and genetic diagnosis of thirteen Japanese patients with hereditary spherocytosis
Source: Hum Genome Var. 2022 Jan 12;9:1. doi: 10.1038/s41439-021-00179-1 (PMC8755803; doi:10.1038/s41439-021-00179-1)
Supplement: Supplementary file 4 — Supplemental Table S3 [file 41439_2021_179_MOESM4_ESM.pdf]

Supplemental Table S3. Distribution of the diease-related variants in the different studies

Symbol	SPH1	SPH2	SPH3	SPH4	SPH5				
Genes	*ANK1*	*SPTB*	*SPTA1*	*SLC4A1*	*EPB42*	Combined	Unknown	others	Total
Inoue et al. 1994	2%	15% (including SPTA1)		32%	6%	36%	9%		
Yawata et al. 2000	7%	0%	0%	20%	45%	0%	28%	0%	
Nakanishi et al. 2001	15	NT	NT	0	0	NT	NT	NT	49
Park et al., 2016	13	12	0	0	0	0	0	0	25
Wang et al., 2018	17	17	0	4	0	0	0	0	38
Lin et al., 2018	2	0	1 (hetero)	0	0	1	3	0	7
Russo et al., 2018	2	5	4	5	0	4	26	28	74
Choi et al., 2019	19	22	0	3	0	6	9	0	59
Xue et al., 2019	3	4	0	2	0	0	0	1	10
van Vuren et al., 2019	23	17	31	13	1	0	10	0	95
Wang et al., 2020	13	10	0	0	0	0	0	0	23
Mansour-Hendili et al., 2020	8	5	0	6	0	1	0	0	20
Xie et al., 2021	13	10	2	3	0	0	0	0	28
This study	6	4	1	1	1	0	0	0	13

NT: not tested
